# Periodontitis Provokes Retinal Neurodegenerative Effects of Metabolic Syndrome: A Cross-Sectional Study

**DOI:** 10.3390/dj12110351

**Published:** 2024-10-31

**Authors:** Hatice Arslan, Nur Yorgancilar, Oguz Kose, Mehmet Gokhan Aslan, Ahmet Altin, Sevda Kurt Bayrakdar, Hatice Yemenoglu, Huseyin Findik, Adnan Yilmaz

**Affiliations:** 1 Department of Periodontology, School of Dentistry, Recep Tayyip Erdogan University, Rize 53100, Turkey; hatice.seviker@erdogan.edu.tr (H.A.); oguz.kose@erdogan.edu.tr (O.K.); hatice.yemenoglu@erdogan.edu.tr (H.Y.); 2 Department of Ophthalmology, School of Medicine, Recep Tayyip Erdogan University, Rize 53100, Turkey; mehmetgokhan.aslan@erdogan.edu.tr (M.G.A.); huseyin.findik@erdogan.edu.tr (H.F.); 3 Department of Periodontology, School of Dentistry, Istanbul Kent University, Istanbul 34433, Turkey; ahmetaltin@outlook.com; 4 Department of Periodontology, School of Dentistry, Eskişehir Osmangazi University, Eskisehir 26040, Turkey; sevda.kurtbayrakdar@ogu.edu.tr; 5 Department of Biochemistry, School of Medicine, Recep Tayyip Erdogan University, Rize 53100, Turkey; adnan.yilmaz@erdogan.edu.tr

**Keywords:** periodontitis, metabolic syndrome, oxidative stress, neurodegeneration, optic coherence tomography

## Abstract

Background: This cross-sectional study aims to investigate the retino-choroidal degenerative effects of periodontitis, metabolic syndrome (Mets), and the combination of these diseases using optical coherence tomography (OCT) measurements. Methods: Ninety-two patients selected according to inclusion criteria were divided into four groups: systemically and periodontally healthy (control), systemically healthy periodontitis (PD), periodontally healthy metabolic syndrome (MetS), and periodontitis and metabolic syndrome combined (PD-MetS). The systemic inflammatory–oxidative effects of periodontitis and MetS were biochemically evaluated using the serum TNF-α level, IL-1β/IL-10 ratio, and oxidative stress index (OSI: TOS/TAS). Retinal (AMT, pRNFLT, and GCL + T) and choroidal (SFCT) morphometric measurements and vascular evaluations (foveal capillary density) were performed via OCT Angio with swept-source technology. Results: Both periodontitis and Mets cause systemic inflammatory stress characterized by significant increases in the IL-1β/IL-10 ratio and OSI (*p* < 0.05). Compared to the control group, the AMT was significantly thinner in the MetS group, the pRNFLT was significantly thinner in the PD-MetS group, and the SFCT was significantly thinner in both groups (*p* < 0.05). The GCL+ was slightly thicker in the Mets groups. (*p* > 0.05) Foveal capillary density did not differ significantly among the groups. (*p* > 0.05). Conclusions: Periodontitis-related inflammatory stress alone causes changes in retinal and subfoveal choroidal thicknesses that are not statistically significant. On the other hand, when combined with Mets, it may significantly provoke the retinal neurodegenerative effects of this disease.

## 1. Introduction

Periodontitis is an inflammatory oral disease that destroys tooth-supporting tissues as a result of complex interactions between periodontal bacteria and host response [1]. However, the current evidence has clearly shown that this disease does not only have local effects such as asthetic and phonetic problems caused by the loss of teeth and loss of chewing function [1,2]. There are keystone pathogens that play a role in this destruction. Red complex bacteria, including *Porphyromonas gingivalis*, *Treponema denticola,* and *Tannerella forsythia,* have been associated with the pathogenesis of periodontitis. Furthermore, periodontitis is thought to result from a dysbiotic change in the periodontal microbiota, with a relative increase in these bacteria. The altered microbiota may be sufficient to initiate this chronic, inflammatory disease [3]. Periodontopathogens, their various virulence factors, and the host response-induced increase of pro-inflammatory mediators and reactive oxygen derivatives expose many vital organs to inflammatory stress through the hematogenous route [4]. In this context, periodontitis can also be defined as a chronic, systemic, subclinically infectious–inflammatory disease. There is strong evidence in the current literature that periodontitis has significant effects on diseases where inflammation plays a central role in their pathogenesis, such as atherosclerotic vascular diseases, diabetes mellitus (DM), Alzheimer’s, reproductive system diseases, and chronic renal failure [2,4,5]. Furthermore, the effects of systemic inflammatory stress associated with periodontitis on metabolic diseases such as non-alcoholic fatty liver disease [6], obesity [7], and metabolic syndrome (Mets) [5,8,9,10], as well as ocular diseases [11,12], are among the current issues of periodontology.

Mets, known as syndrome X, is an entity that is the sum of metabolic disorders such as the increased risk of cardiovascular disease, lipid metabolism disorder, insulin dysregulation, obesity, and high blood pressure, and it is a common disease on a global scale [13]. Similar to periodontitis, the aforementioned components of MetS cause an increase in levels of proinflammatory cytokines such as tumor necrosis factor (TNF)-α, interleukin (IL)-1β, IL-6, and oxidative stress (OS) [8,13,14,15,16]. The current evidence suggests that these two common diseases that share common pathological pathways are characterized by low-grade inflammation and, influenced by genetic and environmental factors, may modify each other’s effects [8,10,13,16].

Systemic chronic inflammatory stress plays a central role in ocular diseases such as cataracts, diabetic retinopathy (DR), glaucoma, and age-related macular degeneration (AMD) [17,18,19,20,21]. OS-related apoptosis and the degeneration of retinal ganglion cells (RGCs) are important common pathologies for retinal diseases [18,21,22,23,24,25]. In addition, inflammatory stress has been shown to impair choroidal vascular support [26,27].

To the best of our knowledge, although there are many clinical studies [19,26,27,28] on the possible effects of Mets on retino-choroidal layers, there is a limited amount of evidence from an animal study [11] on the effects of periodontitis. Moreover, there are no studies on the retinal degenerative effects of the combined presence of periodontitis and Mets. In this context, the present study is unique and based on the hypothesis that the alone or combined presence of periodontitis and Mets may provoke retino-choroidal degenerative changes through systemic inflammatory stress.

## 2. Materials and Methods

### 2.1. Ethical Approval

This cross-sectional study was carried out between May and October 2022 on 92 participants (47 females, 45 males) between the ages of 18 and 65 who applied to the Department of Ophthalmology of the Faculty of Medicine of Recep Tayyip Erdoğan University (RTEU). All participants were selected according to the criteria stated below. The study was approved by the RTEU Faculty of Medicine, Non-Interventional Clinical Research Local Ethics Committee (2022/157) and was performed following the guidelines of the Declaration of the World Medical Association 1975 in Helsinki as revised in 2000. Participants were informed about the study and their verbal and written consent were obtained.

### 2.2. Study Groups

Patients who met the inclusion criteria were assigned to 4 groups of 23 participants: the systemically and periodontally healthy (control) group, systemically healthy periodontitis (PD) group, periodontally healthy metabolic syndrome (MetS) group, and periodontitis and metabolic syndrome combined (PD-MetS) group.

Individuals with stage III/IV, grade C periodontitis according to the current periodontal and peri-implant disease classification [29] were included in the study. Patients with less than 20 teeth were excluded from the study. The diagnosis of Mets was made by a specialist physician following diagnostic criteria by the International Diabetes Federation (IDF) [30]. Accordingly, obese [waist circumference (WC) ≥ 80 cm for women and ≥94 cm for men], Type 2 DM, and hypertensive patients were included in the study.

Individuals with cancer, any autoimmune, osteoporotic, active infectious disease (acute hepatitis, tuberculosis, AIDS), vitreoretinal, optic nerve, or choroidal vascular disease, acute and chronic ocular diseases such as cataract, glaucoma, macular degeneration, uveitis, Behçet’s, scleritis, a history of refractive or intraocular surgery, immunosuppressive, oral contraceptive, bisphosphonate, and antioxidant drug use, pregnancy–lactation, and smoking were not included in the study.

### 2.3. Sample Size Calculation

A power analysis was performed to calculate the minimum sample size required. The sample size was established considering four groups, an effect size of 0.30 for probing pocket depth (PPD) (which represents the primary outcome variable), an expected standard deviation of 0.5 [31], a two-sided significance level of 0.05, and a power of 80%. It was determined that approximately 23 patients per group would be needed.

### 2.4. Examiner Calibration

Calibration was performed on 10 patients (5 periodontally healthy and 5 with periodontitis), who were not included in the study. A single examiner (H.A.) was trained and calibrated for measurements of the clinical periodontal parameters in two different periods (baseline and 10th day). The intraclass correlation (IC) was evaluated with an IC of 0.9902, and *p* < 0.0001 was considered an excellent replicability.

### 2.5. Clinical Periodontal Measurements

Clinical periodontal status was evaluated by plaque index (PI) [32], gingival index (GI) [32], bleeding index (BOP) [33], PPD, and *clinical attachment level* (CAL). PPD was measured as the distance between the deepest point of the sulcus (or pocket) and the gingival margin, and CAL was measured as the distance between the base of the pocket and the *cementoenamel junction*. PPD and CAL were measured at six different points (*mesio-buccal*, *mid-buccal*, *disto-buccal*, *mesio-lingual*, *mid-lingual*, and *disto-lingual*) in each tooth, and other index scores were taken at four different points (*mesial*, *distal*, *vestibular*, and *palatinal*). Measurements were performed by a periodontist (H.A.) using a Williams probe (Hu-Friedy, Chicago, IL, USA).

### 2.6. Sample Collection and Biochemical Analysis

Immediately following the clinical periodontal measurements, approximately 16 cc of venous blood was taken from the *antecubital fossa* region of the left arm of each patient for biochemical analysis. The samples were kept at room temperature for 30 min and centrifuged (Centrifuge Machine, Elektro-mag, Istanbul, Turkey) at 3000× *g* for 15 min, and the serum samples were obtained. The samples were placed into Eppendorf tubes and stored at −80 °C (Thermo Fisher Scientific, Waltham, MA, USA) until the day of biochemical analysis.

Twenty-four hours before the analysis, the samples were taken out from −80 °C storage and left to thaw gradually (first −20 °C, then +4 °C). On the day of analysis, they were taken to room temperature (18–25 °C) and vortexed to obtain homogeneous preparations. Serum IL1-β, TNF-α, and IL-10 levels were measured via the immunosorbent assay (ELISA) method using human-specific kits (Elabscience Biotechnology Co., Houston, TX, USA) according to the kit manufacturer’s instructions. The results were calculated as pg/mL.

Serum total antioxidant status (TAS) and total oxidant status (TOS) levels were measured by a spectrophotometric method using specific kits (Rel Assay Diagnostics, Gaziantep, Turkey) [34,35]. The results were expressed in μmol H_2_O_2_Equiv./L. The oxidative stress index was calculated with the formula (OSI) = [(TOS, μmol/L)/(TAS, mmol Trolox equivalent/L) × 100] [35].

### 2.7. Optical Coherence Tomographic Measurements

Retinal and choroidal imaging and measurements were performed with optical coherence tomography (SS-OCT) (DRI OCT Triton; Topcon Corporation, Tokyo, Japonya), with swept-source (SS) technology and non-invasive OCT angiography (SS-OCT Angio) (DRI OCT Triton; Topcon Corporation, Tokyo, Japan). All measurements were made by a single investigator (G.A.), and only images with an above-average signal strength index were included in the study. Images with motion artifacts were excluded from the analysis. The OCT and OCT Angio were performed in the morning. For every parameter, the average of left and right measurements was obtained.

**Figure 1:**
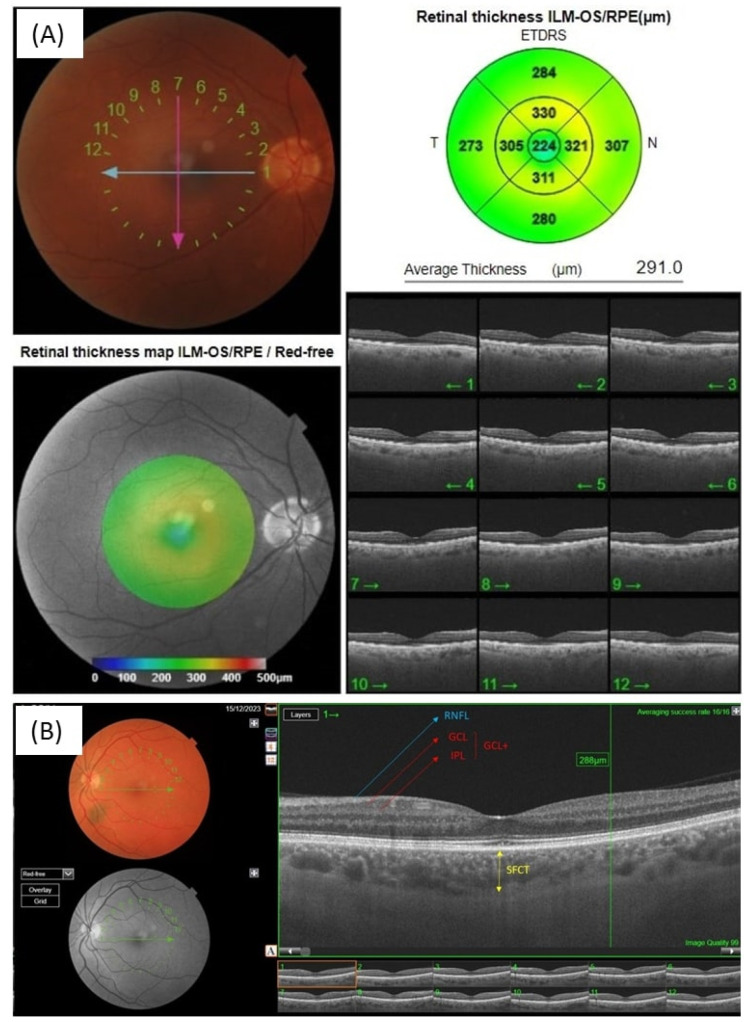
OCT analysis. (**A**) AMD measurement, (**B**) pRNFLT, GCL + T, and SFCT measurements.

**Figure 2:**
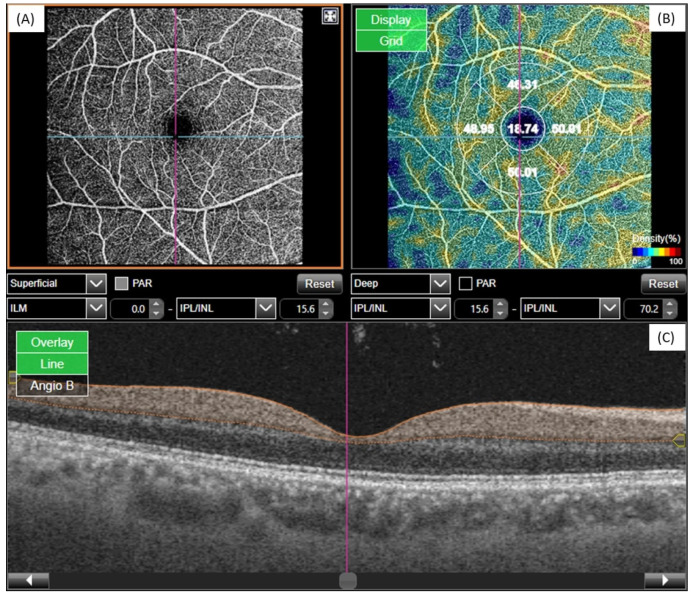
FCD measurements. (**A**) Superficial vessels, (**B**) foveal and parafoveal capillar density values, (**C**) view of the foveal area in one of the SS-OCT sections.

In this study, average macular thickness (AMT) (Figure 1A), peripapillary retinal nerve fiber layer thickness (pRNFLT) (Figure 1B), GCL + T (the interface between the nerve fiber layer and ganglion cell layer to the interface between the inner plexiform layer and inner nucleer layer), subfoveal choroidal thickness (SFCT, the distance between the basement membrane of the retinal pigment epithelium and the choroidoscleral interface) (Figure 1B), and foveal capillary density (FCD) (Figure 2) were selected for analysis.

The three-dimensional (3D) wide protocol was used to evaluate the retina and choroid for all subjects. This protocol includes a wide scanning range that focuses both on the macular (Early Treatment Diabetic Retinopathy Study [ETDRS] scan) and the peripapillary (TSNIT: *Temporal–Superior–Nasal–Inferior–Temporal*) area. The macula was divided into three rings with diameters of 1 mm (center), 3 mm (inner), and 6 mm (outer). The inner and outer rings were divided into four quadrants: *temporal*, *superior*, *nasal*, and *inferior*. The thickness of each layer of the retina and choroid was analyzed using the built-in software (Imagenet 6, version: 1.32) of the SS-OCT device (version: 1.1.9) (Figure 1A).

The pRNFLT was obtained from a 3.4 mm diameter circle centered on an optical disc (6 mm × 6 mm scan, 512 A-scan×256 B-scan). The pRNFLTs in the *temporal*, *nasal*, *inferior*, and *superior* quadrants were obtained with the system’s built-in automatic segmentation algorithm, and the mean pRNFLT was recorded for analysis (Figure 1B).

Macular evaluation using OCT provides measurements of the macular area displayed as an ETDRS grid (reporting the thickness of nine measured areas). The AMT [6 mm × 6 mm scan; 1024 × 12 (12 radial scan lines consisting of 1024 A-scans)] and GCL + T (6 mm × 6 mm scan; 512 × 128) were obtained by the 3D macular scanning protocol. The SFCT was also measured with the automatic software (Imagenet 6, version: 1.32) of the device (version: 1.1.9) (Figure 1A,B).

In SS-OCT Angio measurements, vessel density analysis was performed in a superficial retinal layer (Figure 2A). A 3D scan (6.0 × 6.0 mm–320 × 320) area map was used in all patients. The reference plane for SVD was defined as the area from the inner limiting membrane (ILM) to 15.6 µm below the inner plexiform layer (IPL). Vessel density measurement was made in the foveal (1 mm diameter central region) and parafoveal (1–3 mm diameter ring) area. The parafoveal regions were divided into 4 sections of 90 degrees each (*nasal*, *inferior*, *superior*, and *temporal* sectors) (Figure 2B). The mean foveal vessel density values were used for statistical analysis (Figure 2A–C).

### 2.8. Statistical Analysis

SPSS (IBM, Chicago, IL, USA) (version 23.0) software on Windows was used for the statistical analysis. The normal distribution was checked by the Shapiro–Wilk test and by examining histogram graphs. In cases of normal distribution, the One-Way ANOVA test was applied to examine the difference between the means of three or more independent groups; in cases where the normal distribution assumption was not met, the Kruskal–Wallis test was performed. Post hoc Bonferroni correction was applied to the significance values for multiple tests. *p* < 0.05 was considered statistically significant.

## 3. Results

### 3.1. Demographic Results

There was no significant difference between and within the groups in terms of sex. The ages of the participants ranged from 28 to 59 years, and there was no significant difference between the groups in terms of mean age. Values for the diagnostic criteria for obesity [waist circumference (WC), body mass index (BMI)] and diabetes (HbA1c) were significantly higher in the MetS groups compared to the control and PD groups. (*p* < 0.05). (Table 1)

### 3.2. Clinical Periodontal Results

**Figure 3:**
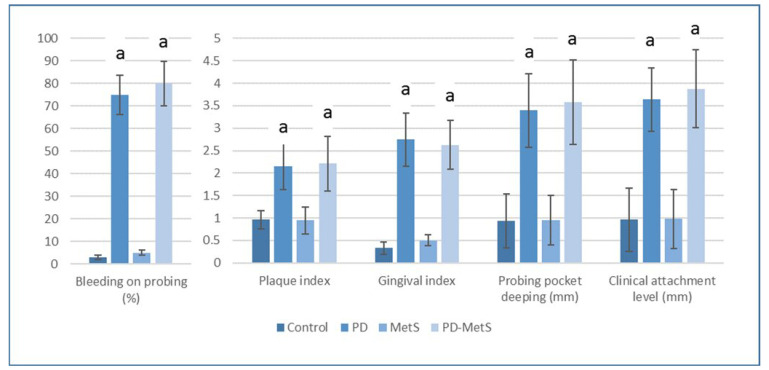
Intergroup comparisons of clinical periodontal parameters. PD, periodontitis group; MetS, metabolic syndrome group; PD-MetS, periodontitis–metabolic syndrome group. Data are expressed as mean ± SD. (a) Compared to control and MetS groups; statistically significant difference (*p* < 0.05).

All clinical periodontal parameters were significantly higher in periodontitis groups (PD and PD-MetS) compared to periodontal healthy groups (control and MetS). (*p* < 0.05) On the other hand, no significant difference was found in in-group evaluations (*p* > 0.05) (Figure 3).

### 3.3. Biochemical Results

**Figure 4:**
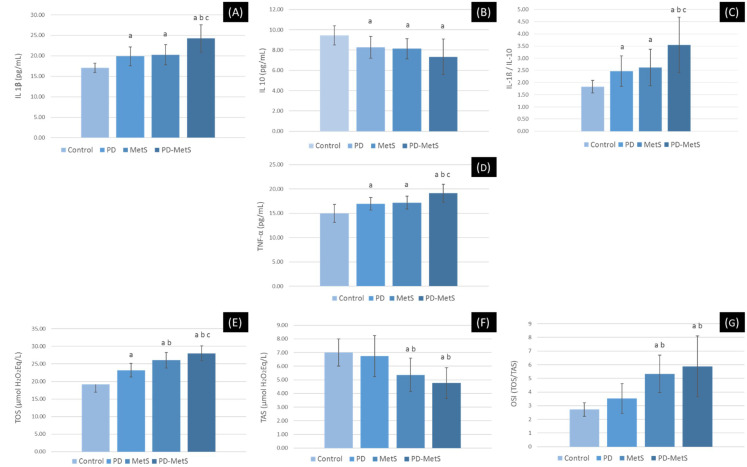
Intergroup comparisons of biochemical parameters. (**A**) Interleukin 1-beta (IL-1ß). (**B**) Interleukin 10 (IL-10). (**C**) IL-1ß/IL-10. (**D**) Tumor necrosis factor-alpha (TNF-α). (**E**) Total oxidant status (TOS). (**F**) Total antioxidant status (TAS). (**G**) Oxidative stress index (OSI). PD, periodontitis group; MetS, metabolic syndrome group; PD-MetS, periodontitis–metabolic syndrome group. Data are expressed as mean ± SD. (a) Compared to control group; (b) compared to PD group; (c) compared to MetS group; statistically significant difference (*p* < 0.05).

Serum TNF-α, IL-1β, and IL-1β/IL-10 ratio levels were significantly higher in all other groups compared to the control group; and were significantly higher in the PD-MetS group compared to the PD and MetS groups. (*p* < 0.05). By contrast, IL-10 was significantly lower in all other groups compared to the control group (*p* < 0.05) (Figure 4A–D).

The serum TOS level was significantly higher in all other groups compared to the control group; significantly higher in the two MetS groups compared to the periodontitis group; and significantly higher in the PD-MetS group compared to the MetS group (*p* < 0.05). Conversely, TAS levels were significantly lower in the MetS groups compared to the systemically healthy groups (*p* < 0.05). In contrast to the TAS findings and partially compatible with the TOS levels, the OSI levels were found to be significantly higher in the MetS groups compared to the other groups (*p* < 0.05) (Figure 4E–G).

### 3.4. OCT Measurements

**Figure 5:**
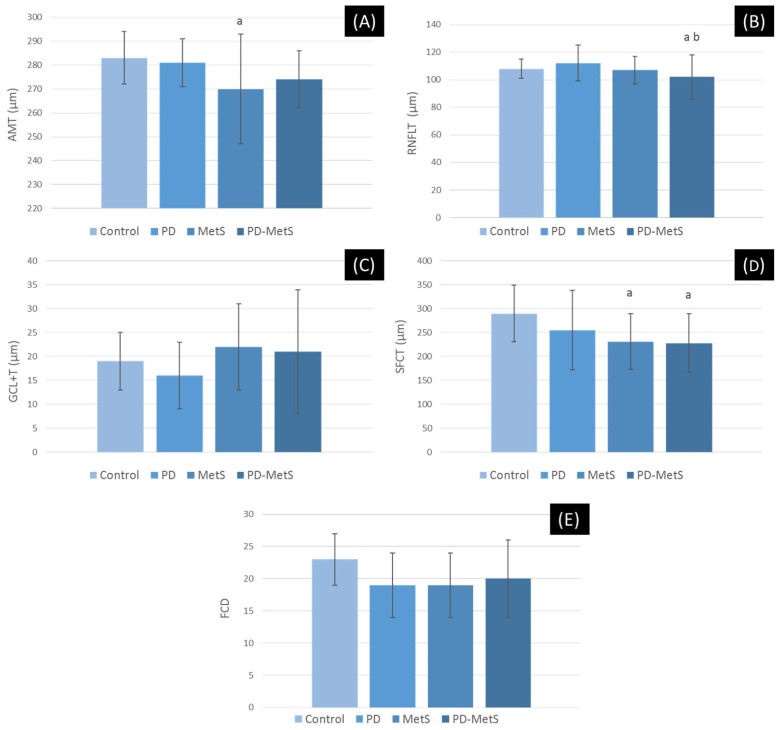
Intergroup comparisons of OPT and OPT-Angio measurements. (**A**) Average macular thickness (AMT). (**B**) Retinal nerve fiber layer thickness (PRFLT). (**C**) Total thickness of ganglion cell and inner plexiform layers (GCL + T). (**D**) Subfoveal choroideal thickness (SFCT). (**E**) Foveal capillary density (FCD). PD, periodontitis group; MetS, metabolic syndrome group; PD-MetS, periodontitis–metabolic syndrome group. Data are expressed as mean ± SD. (a) Compared to control group; (b) compared to PD group; statistically significant difference (*p* < 0.05).

The AMT was lower in all groups compared to the control group, and macular thinning was statistically significant only in the MetS group (*p* < 0.05). The pRNFLT was significantly thinner only in the PD-MetS group, compared to the control and periodontitis groups. The SFCT was significantly lower in the two MetS groups compared to the other groups. Compared to the healthy group, the GCL+ was slightly thinner in the periodontitis group and slightly thicker in the two MetS groups (*p* > 0.05). The decrease in FCD measurements by SS-OCT Angio, in all groups compared to the control group, was not statistically significant. (*p* > 0.05) (Figure 5A–E)

## 4. Discussion

When our ocular morphometric findings are evaluated in general, MetS causes a more significant thinning of the retinal and choroid layers, compared to periodontitis. Furthermore, periodontitis can significantly provoke MetS-associated retinal neurodegeneration. On OCT angiography, there was no significant difference between the groups in terms of FCD.

In the present study, we investigated systemic inflammatory status with TNF-α, IL-1β, and IL-10, the inflammatory cytokines with a central role in the pathogenesis of both diseases [8]. OS was analyzed with TOS and TAS kits, which were introduced by Erel [34,35] and reflect the final oxidative state and antioxidant capacity practically. OSI is the ratio of TOS to TAS and reflects the change in OS in an even more practical way [35]. OS is an inflammatory condition that occurs as a result of the disruption of the dynamic interaction between the intense release of reactive oxygen species and the antioxidant mechanisms responsible for their inactivation, in favor of free radicals or against antioxidant defenses. OS can cause vital damage to biomolecules such as membrane lipids, proteins, and DNA, which are vital for tissue homeostasis and cellular functions [36]. Our findings that periodontitis and MetS cause a significant increase in thr serum TNF-α level and IL-1β/IL-10 ratio and that the combined presence of these diseases exacerbates systemic inflammatory stress are compatible with the consensus in previous studies [8,9,14,15,16]. Similarly, the findings of the present study that TOS and OSI increased significantly in the combined presence of both diseases compared to their alone presence support the previous strong evidence [10,37]. Recent studies clearly show that inflammation and OS play a central role in the pathogenesis of retinal degenerative diseases that can result in partial or complete blindness, such as AMD, DR, and glaucoma [17,18,38,39].

OCT is a noninvasive, noncontact, safe, and effective method for retinal morphometric examinations. It is the most sensitive method for measuring retinal thickness in vivo [40]. It may indicate a thinning of the inner layer of the retina long before the symptomatic diagnosis of DR [22,40]. Decreases in macular, pRNFL, and GCL+ thicknesses are considered to be indicators of apoptosis and neurodegeneration in RGCs. Evaluation of our retinal measurements in general shows that MetS caused a more significant thinning in AMT compared to periodontitis, and the combined presence of the two diseases significantly reduced the pRNFLT, especially. Supporting this, New et al. [28] reported that the pRNFL may be more sensitive to MetS-associated neurodegeneration, compared to the macula. In another study, Zarei et al. [19] stated that MetS is independently associated with a reduced RNFLT, i.e., neurodegeneration is implicated in the pathogenesis of MetS. Moreover, obesity, which is one of the key components of MetS, has also been shown to lead to a decrease in pRNFLT in adults [22] and even in young people [20]. We are of the belief that the marked retinal neurodegenerations characterized by pRNFL and macular thinning in the MetS and PD-MetS groups are a result of subclinical systemic inflammation and OS caused by these diseases. RGCs have vital functions in the realization and quality of vision: the connection between photoreceptors and the brain, the processing of visual signals, and the management of visual information [23]. Therefore, RGC dysfunction or degeneration is a fundamental pathology for many ocular diseases associated with loss of vision [24]. Tezel [21] reported that intraocular OS not only causes direct damage to RGCs but also indirectly induces apoptosis in these cells through caspase activation. Supporting this, in an in vitro study, the suppression of free radicals has been shown to protect RGCs from apoptosis [25]. Moreover, OS markers such as malondialdehyde and 8-hydroxy-2′-deoxyguanosine are thought to be potential biomarkers for the incidence and/or progression of AMD [38].

Interestingly, we also found a slight thickening of the GCL+, especially in the MetS groups. This thickening may be related to cellular swelling as a result of hypoxia [41] triggered by TNF-α and IL-1β. In other studies measuring GCL thickness by OCT, Panon et al. [41] found a slight increase in obese patients, while Polat et al. [42] found a slight decrease in MetS patients. The differences between the findings may be related to the duration of exposure to inflammation and concomitant inflammatory diseases.

To the best of our knowledge, there is no clinical evidence, except for the present study, that morphometrically examines the possible neurodegenerative effects of periodontitis on the retinal and choroidal layers. In a recent animal study [11], it was determined that ligature and *P. gingivalis* applications in an experimental AMD model caused a significant decrease in total retinal thickness in OCT. Lindner et al. [43], on the other hand, found a significant relationship between periodontitis and mild and moderate DR rather than severe and proliferative DR in study groups with a mean age of over 60 years. Statistically non-significant changes in AMT and RNFLT in patients with periodontitis may be related to the mean age (44 ± 9 years) of our study groups, which can be considered relatively low for ocular diseases. Epidemiological studies show that retinal degenerative diseases usually occur from the age of 60 [44]. The clinical aspects of the retinal degenerative effect of periodontitis will likely be more evident at older ages. However, the significant RNFLT thinning in the PD-MetS group indicates that periodontitis significantly provokes MetS-associated retinal neurodegeneration and apoptosis.

We evaluated the effects of periodontitis and MetS on foveal and subfoveal vascular structures by FCD and SFCT analysis. We found slight decreases in FCD in SS-OCT Angio in all disease groups compared to healthy patients. In contrast, the SFCT was slightly thinner in the periodontitis group and significantly thinner in the MetS and PD-MetS groups. The choroidal layer of the eye is one of the tissues with the highest blood flow in the human body. It has vital functions in retinal physiology, such as supplying oxygen and nutrients to the retinal layers and removing residues. Therefore, choroid physiology has an important role in the emergence and progression of retinal diseases [45]. In support of our findings, MetS [26] and obesity [27] have been shown to cause a significant decrease in SFCT. In this context, the significant thinning, especially in the MetS groups, suggests that one of the causes of periodontitis and the aforementioned MetS-related retinal neurodegenerations may be the weakening of vascular support from the choroid. Our findings that SFCT is reduced at a statistically non-significant level in patients with periodontitis are partially consistent with a single study investigating the effects of periodontitis on the sub-retinal layer. In their valuable study, Karesvuo et al. [12] found a significant relationship between high active matrix metalloproteinase-8 levels and the sub-retinal fibrosis of the fovea in AMD patients, which is a clinical manifestation of the later period of wet AMD.

This cross-sectional study has some important limitations. First, the duration of exposure to obesity, DM, and hypertension was unknown, especially in patients with MetS. Secondly, thinning of the retinal layers was observed, especially from the age of 50–60 [44]. Mild retinal thinning, especially in the periodontitis group, might be related to the lower age range of our participants. Lastly, MetS patients in the study were individuals who were already taking antidiabetic medications. Previous studies [46,47] have shown that the use of metformin, insulin, and low-dose statins may limit neurodegeneration. Our retinal morphometric measurements, which were not at a level of statistical significance, might be the result of the drugs used. In this context, it is clear that new studies are needed to examine the possible retino-choroidal degenerative effects of periodontitis alone or in combination with MetS in larger standardized groups of participants with different age ranges.

## 5. Conclusions

OCT measurements showed that, in the 40–50 age range, periodontitis caused limited retino-choroidal structural changes. However, when combined with MetS, it exacerbated MetS-associated retinal neurodegenerative effects. Retinal and choroidal thinning may be the result of apoptotic damage to neural and vascular cells provoked by chronic inflammation and OS.

## Figures and Tables

**Table 1:** Demographic findings.

Parametres		Control	PD	MetS	PD-MetS	*p* Value
Sex	Female	11 (47.8)	11 (47.8)	13 (56.5)	12 (52.2)	0.224
	Male	12(52.2)	12 (52.2)	10 (43.5)	11 (47.8)	
Age		43 ± 8	44 ± 9	45 ± 8	44 ± 6	0.612
WC (cm)		77 ± 10	81 ± 9	94 ± 7 * ^†^	95 ± 6 * ^†^	<0.001
BMI (kg/m^2^)		21.6 ± 4	21.8 ± 1.7	27.7 ± 2.3 * ^†^	30.7 ± 3.5 * ^†^	<0.001
HbA1c (%)		5.13 ± 0.58	5.22 ± 0.54	7.51 ± 0.59 * ^†^	7.45 ± 0.57 * ^†^	<0.001

Data are expressed as number (%) and mean ± SD. *p* values were obtained from One-Way ANOVA or Kruskal–Wallis analyses. * Statistical significance between groups compared to control group. ^†^ Statistical significance between groups compared to PD group. WC, waist circumference; BMI, body mass index.

## Data Availability

The original contributions presented in this study are included in the article. Further inquiries can be directed to the corresponding author.
